# A Low Psoas Muscle Index before Treatment Can Predict a Poorer Prognosis in Advanced Bladder Cancer Patients Who Receive Gemcitabine and Nedaplatin Therapy

**DOI:** 10.1155/2017/7981549

**Published:** 2017-04-13

**Authors:** Ryo Kasahara, Takashi Kawahara, Shinji Ohtake, Yoko Saitoh, Sohgo Tsutsumi, Jun-ichi Teranishi, Yasuhide Miyoshi, Noboru Nakaigawa, Masahiro Yao, Kazuki Kobayashi, Hiroji Uemura

**Affiliations:** ^1^Departments of Urology and Renal Transportation, Yokohama City University Medical Center, Yokohama, Japan; ^2^Department of Urology, Yokosuka Kyosai Hospital, Yokosuka, Japan; ^3^Department of Urology, Yokohama City University Graduate School of Medicine, Yokohama, Japan

## Abstract

*Introduction*. Gemcitabine and cisplatin (GC) is a gold-standard first-line systemic chemotherapy for advanced urothelial carcinoma (UC). However, it may cause severe adverse effects such as renal toxicity, gastrointestinal toxicity, and neurotoxicity. Sarcopenia is the age-related loss of skeletal muscle mass. A correlation between sarcopenia and the oncological prognosis has been reported. In UC, several studies have noted that patients with sarcopenia had a greater incidence of complications and worse survival after radical cystectomy or chemotherapy. Our institute introduced gemcitabine and nedaplatin (GN) for UC patients with renal failure. We investigated whether the presence of sarcopenia predicted the prognosis of patients with advanced UC who were treated by GN chemotherapy.* Methods*. A total of 27 patients (male, *n* = 21; female, *n* = 6) received GN therapy for metastatic UC from 2005 to 2016. The institutional review board of Yokohama City University Hospital approved this study. The psoas muscle index (PMI, cm^2^/m^2^) was calculated using this formula: right psoas muscle area (cm^2^)/the square of the body height (m^2^). The overall survival (OS) of the high PMI group (male: ≥2.49, female: ≥2.07) and low PMI group (male: <2.49, female: <2.07) was compared.* Results*. Kaplan-Meier survival curves and a log-rank test revealed that the high PMI group had significantly better OS than the low PMI group (*p* = 0.015). The mean survival of the high and low PMI groups was 561 days and 223 days, respectively.* Conclusions*. In the present study, we revealed that sarcopenia (a low psoas muscle volume) might be a predictive factor for poorer overall survival in patients with advanced urothelial carcinoma who are undergoing GN chemotherapy.

## 1. Introduction

Gemcitabine and cisplatin (GC) is a gold-standard first-line systemic chemotherapy for advanced urothelial carcinoma (UC). However, it may cause severe adverse effects such as renal toxicity, gastrointestinal toxicity, and neurotoxicity. Its nephrotoxic characteristic makes it particularly unsuitable for advanced UC patients because they may have either age-associated or postsurgical renal dysfunction. Instead, nedaplatin, a second-generation platinum complex with lower renal and gastrointestinal toxicity than cisplatin, can be used for patients with a marginal renal function [1–4].

Sarcopenia is the term used to describe the age-related loss of skeletal muscle mass [5]. A correlation between sarcopenia and the oncological prognosis has been reported in malignant melanoma, breast cancer, hepatocellular carcinoma, gastric cancer, and pancreatic cancer [6–10]. In urothelial carcinoma, several studies have noted that patients with sarcopenia have a greater incidence of complications and worse survival after radical cystectomy or chemotherapy [11–13]. Taguchi et al. reported that sarcopenia is associated with a significantly worse prognosis in metastatic UC patients who receive systemic chemotherapy [14]. However, no studies have reported whether sarcopenia can be used as a prognostic factor for metastatic UC patients undergoing gemcitabine and nedaplatin (GN) chemotherapy. Because our institutions have used GN therapy to treat urothelial carcinoma, we investigated whether sarcopenia could predict the prognosis of patients with advanced UC who receive GN chemotherapy.

## 2. Materials and Methods

### 2.1. Patients

A total of 27 patients (male, *n* = 21; female, *n* = 6) received GN therapy for metastatic UC at Yokohama City University Hospital (Yokohama, Japan) from 2005 to 2016. All of the patients were Asian (Japanese). The institutional review board of Yokohama City University Hospital approved this study. The patients' characteristics are summarized in Tables 1 and 2. Among these patients, 8 (29.6%) underwent radical cystectomy and 8 (29.6%) underwent nephroureterectomy prior to GN therapy. The mean age was 61.9 years (range: 38–73). The mean creatinine clearance (CCr) was 70.1 ml/min (range: 40.0–109.0). Twelve patients had kidney dysfunction (CCr ≤ 12 ml/min.), while 15 patients had an adequate renal function (CCr ≥ 60 ml/min.).

### 2.2. Drug Administration and the Evaluation of Responses

Patients received gemcitabine 1,000 mg/m^2^ on days 1 and 8 plus nedaplatin 80 or 100 mg/m^2^ on day 1. Dose modification was allowed depending on the patient's general condition, CCr, and bone marrow suppression status. All patients received at least 2 cycles of GN chemotherapy. The tumor response was assessed after 2 cycles of GN chemotherapy according to the Response Evaluation Criteria in Solid Tumor (RECIST). Adverse effects were assessed according to the Common Terminology Criteria for Adverse Events (CTCAE) version 4.0.

### 2.3. Clinical Assessments

The area of the right psoas muscle on axial CT was calculated at the level of the L3 before GN therapy. We previously examined the psoas muscle volume and surgical complications using right/left and L3 level/umbilicus. As the right psoas muscle volume showed the highest sensitivity; we used the right L3 level psoas volume for further assessments. The psoas muscle index (PMI, cm^2^/m^2^) was calculated using this formula: right psoas muscle area (cm^2^)/the square of the body height (m^2^). The overall survival (OS) of the high PMI group (male: ≥2.49, female: ≥2.07) and low PMI group (male: <2.49, female: <2.07) was compared. The raw data of psoas muscle area and body height are attached as Supplementary Material available online at https://doi.org/10.1155/2017/7981549.

### 2.4. Cut-Off Values

Due to the differences in the PMI values of male and female patients, we set the cut-off point for each gender. The median cut-off PMI values for males and females were 2.49 and 2.07, respectively (*p* = 0.026). There were no statistically significant differences in the baseline characteristics of the high and low PMI groups.

### 2.5. Statistical Analysis

The patients' characteristics and preoperative factors were analyzed using chi-squared test. Kaplan-Meier product limit estimation was used to estimate the OS. The survival duration was defined as the time between the day of starting the 1st course of GN therapy and death. The log-rank test was used for comparisons. *p* values of <0.05 were considered to indicate statistical significance.

## 3. Results

### 3.1. The Follow-Up Period, Tumor Responses, and the Incidence of Adverse Effects

The median and mean follow-up period were 14.0 and 13.7 months (13.7 ± 8.12). Thirteen patients showed a complete response (CR) or partial response (PR), and 14 patients had stable disease (SD) or progressive disease (PD) after 2 courses of GN therapy. The frequent adverse effects (CTCAE Grade ≥ 3) were neutropenia (*n* = 25; 92.6%) and thrombocytopenia (*n* = 17; 63.0%). Granulocyte-colony stimulating factor agents were used in 21 cases (77.8%).

### 3.2. The Correlation of the PMI and OS

**Figure 1:**
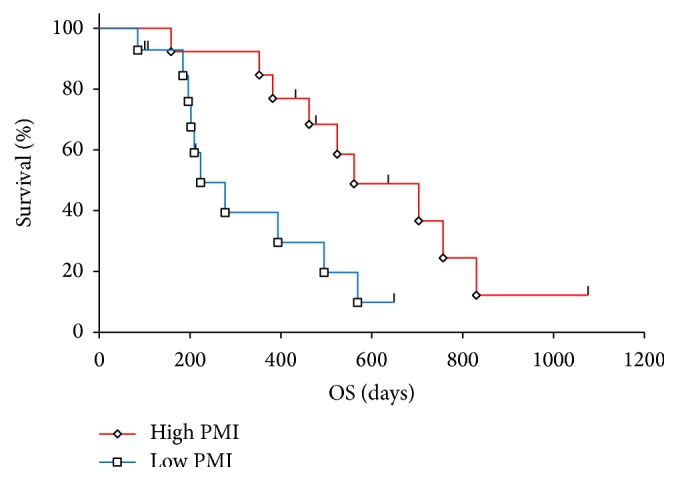
Overall survival in patients with high and low PMIs.

The OS of patients with high and low PMI values was compared. The Kaplan-Meier curves and log-rank test results revealed that the OS of the high PMI group was significantly better than that of the low PMI (*p* = 0.0150, Figure 1.). The mean survival duration in the high and low PMI groups was 561 days and 223 days, respectively.

### 3.3. Adverse Effects

Neutropenia and thrombocytopenia (Grade 3 or 4) were observed in 25 (92.6%) and 21 (77.8%) patients, respectively. One patient had grade 3 appetite loss. The rates of adverse effects in the high and low PMI groups did not differ to a statistically significant extent.

## 4. Discussion

At present, GC therapy is a gold-standard treatment for advanced UC. Cisplatin binds to serum proteins easily; thus, in comparison to nedaplatin or carboplatin, a smaller portion of the injected platinum is excreted into the urine [15]. It is thought that this characteristic of cisplatin is responsible for the severe adverse effects observed in cisplatin-based chemotherapy. Thus, other platinum complexes can be used to reduce the toxicity of chemotherapy. Carboplatin, a cisplatin analogue, shows improved toxicity and favorable anticancer efficacy. The response rate in patients with upper urinary tract UC is reported to be 18.4% [16]. Moreover, nedaplatin, a second-generation platinum complex, has been developed. Nedaplatin is associated with less renal toxicity and a lower incidence of gastrointestinal complications than cisplatin [1, 3]. Matsumoto et al. reported that GN therapy had greater efficacy against lung cancer in vivo than GC or gemcitabine and carboplatin [4]. In our institution, based on these merits, we have administered nedaplatin-based chemotherapy to patients with advanced UC and have demonstrated good results [1, 2].

Gemcitabine and platinum complex-based chemotherapy has improved the survival of patients with advanced UC. However, some patients show aggressive disease progression and die within a few months. Hence, we need reliable and convenient predictors of patients' survival. Sarcopenia refers to the degenerative loss of skeletal muscle associated with aging, cachexia, and frailty—these conditions, termed geriatric syndrome, result from the age-related cumulative decline in the function of multiple organs [5]. Recently, sarcopenia has been reported as a predictive factor for various malignancies. For instance, sarcopenia is reported to be a useful predictor of cancer survival and the perioperative outcomes of melanoma, breast cancer, hepatocellular carcinoma, gastric cancer, and pancreatic cancer [6–10].

In UC, several studies have reported that sarcopenia is associated with an increased risk of perioperative complications and that it is correlated with a poor prognosis after radical cystectomy [11–13]. Taguchi et al. reported that sarcopenia was an independent predictor of a poor prognosis in patients with metastatic UC who underwent first-line systemic chemotherapy [14].

In previous studies on sarcopenia, dual-energy X-ray absorptiometry and a bioelectrical impedance analysis were used to calculate the skeletal muscle volume. However, these examinations are not performed in the routine medical care of patients with malignancies. We therefore used standard axial CT to calculate the psoas muscle volume. We then revealed that the PMI (calculated by the right-side psoas muscle area and height) can predict a patient's overall survival. One limitation of this study is that it included both surgery and nonsurgery patients. Cystectomy and nephroureterectomy are important factors influencing both the OS and PFS. Our institution allocated patients to receive cystectomy or nephroureterectomy based on their age and performance status. Sarcopenia might have therefore influenced the outcomes of cystectomy and nephroureterectomy. Further studies are needed to corroborate our findings.

In the present study, our findings revealed that sarcopenia (a low psoas muscle volume) may be a predictive factor for poorer overall survival in patients with advanced urothelial carcinoma undergoing GN chemotherapy.

## Supplementary Material

Supplementary Table 1: Patients' height and psoas muscle area.

## Figures and Tables

**Table 1:** The clinical characteristics of the patients.

	Total (*n* = 27)	High PMI (*n* = 13)	Low PMI (*n* = 14)	*p* value
Age (years)				
<65	13 (48.1%)	7 (53.8%)	6 (42.9%)	0.568
≥65	14 (51.9%)	6 (46.2%)	8 (57.1%)	
Gender				
Female	6 (22.2%)	3 (23.1%)	3 (21.4%)	0.918
Male	21 (77.8%)	10 (76.9%)	11 (78.6%)	
Creatinine clearance (mL/min.)				
<60	12 (44.4%)	6 (46.2%)	6 (42.9%)	0.8632
≥60	15 (55.6%)	7 (53.8%)	8 (57.1%)	
Cystectomy or nephroureterectomy				
Yes	16 (59.3%)	10 (76.9%)	6 (42.9%)	0.0719
No	11 (40.7%)	3 (23.1%)	8 (57.1%)	
Response after 2 courses of GN therapy				
CR and PR	13 (48.1%)	10 (76.9%)	3 (21.4%)	0.0719
SD and PD	14 (51.9%)	6 (46.2%)	8 (57.1%)	
Adverse effects (grade ≥ 3)				
Neutropenia	25 (92.6%)	12 (92.3%)	13 (92.9%)	0.9566
Thrombocytopenia	17 (63.0%)	8 (61.5%)	9 (64.3%)	0.6802
Appetite loss or nausea	1 (3.7%)	1 (7.7%)	0 (0%)	0.2903

**Table 2:** Locations of metastases and responses after two courses of GN therapy.

	CR·PR	SD	PD	Total
Lymph node	13 (59%)	7	2	22
Lung	4 (57%)	1	2	7
Liver	2 (67%)	1	0	3
Bone	0 (0.0%)	2	0	2
Local recurrence	1 (17%)	5	0	6
Urethra	0 (0.0%)	1	0	1

## Data Access

Due to ethical restrictions, the raw data underlying this paper is available upon request to the corresponding author.
