# Supplementary material for: A Low Psoas Muscle Index before Treatment Can Predict a Poorer Prognosis in Advanced Bladder Cancer Patients Who Receive Gemcitabine and Nedaplatin Therapy
Source: Biomed Res Int. 2017 Apr 13;2017:7981549. doi: 10.1155/2017/7981549 (PMC5406717; doi:10.1155/2017/7981549)
Supplement: Supplementary file 1 — Supplementary Table 1: Patients' height and psoas muscle area. [file 7981549.f1.docx]

Supplementary Table 1: Patients’ height and psoas muscle area

| Patients | Height | L3 Rt | right PMI | High PMI/low PMI |
| --- | --- | --- | --- | --- |
| No. 1 | 167.0 | 5.23 | 1.88 | low PMI |
| No. 2 | 168.3 | 12 | 4.24 | high PMI |
| No. 3 | 173.4 | 6.44 | 2.14 | low PMI |
| No. 4 | 149.5 | 3.93 | 1.76 | low PMI |
| No. 5 | 168.0 | 7.6 | 2.69 | high PMI |
| No. 6 | 156.2 | 8.26 | 3.39 | high PMI |
| No. 7 | 179.0 | 8.98 | 2.80 | high PMI |
| No. 8 | 147.7 | 5.02 | 2.30 | high PMI |
| No. 9 | 171.0 | 6.18 | 2.11 | low PMI |
| No. 10 | 168.5 | 9.53 | 3.36 | high PMI |
| No. 11 | 177.0 | 8.23 | 2.63 | high PMI |
| No. 12 | 167.1 | 6.2 | 2.22 | low PMI |
| No. 13 | 146.6 | 5.12 | 2.38 | high PMI |
| No. 14 | 169.4 | 8.66 | 3.02 | high PMI |
| No. 15 | 159.7 | 6.34 | 2.49 | low PMI |
| No. 16 | 170.0 | 6.21 | 2.15 | low PMI |
| No. 17 | 171.5 | 7.73 | 2.63 | high PMI |
| No. 18 | 169.3 | 7.76 | 2.71 | high PMI |
| No. 19 | 156.5 | 4.55 | 1.86 | low PMI |
| No. 20 | 170.1 | 6.62 | 2.29 | low PMI |
| No. 21 | 160.8 | 5.88 | 2.27 | high PMI |
| No. 22 | 167.5 | 9.88 | 3.52 | high PMI |
| No. 23 | 161.1 | 2.92 | 1.13 | low PMI |
| No. 24 | 154.3 | 3.16 | 1.33 | low PMI |
| No. 25 | 162.8 | 3.65 | 1.38 | low PMI |
| No. 26 | 165.3 | 6.21 | 2.27 | low PMI |
| No. 27 | 165.1 | 6.59 | 2.42 | low PMI |
